# Photon-shot-noise-limited transient absorption soft X-ray spectroscopy at the European XFEL

**DOI:** 10.1107/S1600577523000619

**Published:** 2023-02-20

**Authors:** Loïc Le Guyader, Andrea Eschenlohr, Martin Beye, William Schlotter, Florian Döring, Cammille Carinan, David Hickin, Naman Agarwal, Christine Boeglin, Uwe Bovensiepen, Jens Buck, Robert Carley, Andrea Castoldi, Alessandro D’Elia, Jan-Torben Delitz, Wajid Ehsan, Robin Engel, Florian Erdinger, Hans Fangohr, Peter Fischer, Carlo Fiorini, Alexander Föhlisch, Luca Gelisio, Michael Gensch, Natalia Gerasimova, Rafael Gort, Karsten Hansen, Steffen Hauf, Manuel Izquierdo, Emmanuelle Jal, Ebad Kamil, Suren Karabekyan, Thomas Kluyver, Tim Laarmann, Tobias Lojewski, David Lomidze, Stefano Maffessanti, Talgat Mamyrbayev, Augusto Marcelli, Laurent Mercadier, Giuseppe Mercurio, Piter S. Miedema, Katharina Ollefs, Kai Rossnagel, Benedikt Rösner, Nico Rothenbach, Andrey Samartsev, Justine Schlappa, Kiana Setoodehnia, Gheorghe Sorin Chiuzbaian, Lea Spieker, Christian Stamm, Francesco Stellato, Simone Techert, Martin Teichmann, Monica Turcato, Benjamin Van Kuiken, Heiko Wende, Alexander Yaroslavtsev, Jun Zhu, Serguei Molodtsov, Christian David, Matteo Porro, Andreas Scherz

**Affiliations:** a European XFEL, Holzkoppel 4, 22869 Schenefeld, Germany; b Faculty of Physics and Center for Nanointegration Duisburg-Essen (CENIDE), University Duisburg-Essen, Lotharstrasse 1, 47057 Duisburg, Germany; c Deutsches Elektronen-Synchrotron DESY, Notkestrasse 85, 22607 Hamburg, Germany; d Linear Coherent Light Source, SLAC National Accelerator Lab, 2575 Sand Hill Rd, Menlo Park, CA 94025, USA; e Paul Scherrer Institute, 5232 Villigen PSI, Switzerland; f Université de Strasbourg, CNRS, Institut de Physique et Chimie des Matériaux de Strasbourg, UMR 7504, F-67000 Strasbourg, France; g Politecnico di Milano, Dip. Elettronica, Informazione e Bioingegneria and INFN, Sezione di Milano, Milano, Italy; h IOM-CNR, Laboratorio Nazionale TASC, Basovizza SS-14, km 163.5, 34012 Trieste, Italy; i Institute for Computer Engineering, University of Heidelberg, Mannheim, Germany; j Max-Planck Institute for the Structure and Dynamics of Matter, Luruper Chaussee 149, 22761 Hamburg, Germany; k University of Southampton, Southampton SO17 1BJ, United Kingdom; l Institute for Methods and Instrumentation for Synchrotron Radiation Research (PS-ISRR), Helmholtz-Zentrum Berlin für Materialien und Energie GmbH (HZB), Albert-Einstein Straße 15, 12489 Berlin, Germany; m Institute of Optical Sensor Systems, DLR (German Aerospace Center), Rutherfordstrasse 2, 12489 Berlin, Germany; n Institute of Optics and Atomic Physics, Technische Universität Berlin, Strasse des 17 Juni 135, 10623 Berlin, Germany; o Sorbonne Université, CNRS, Laboratoire de Chimie Physique-Matière et Rayonnement, LCPMR, 75005 Paris, France; p The Hamburg Centre for Ultrafast Imaging CUI, Luruper Chaussee 149, 22761 Hamburg, Germany; q INFN – Laboratori Nazionali di Frascati, via Enrico Fermi 54, 00044 Frascati, Italy; r RICMASS – Rome International Center for Materials Science Superstripes, 00185 Rome, Italy; s Istituto Struttura della Materia, CNR, Via del Fosso del Cavaliere 100, 00133 Rome, Italy; t Institute of Experimental and Applied Physics, Kiel University, 24098 Kiel, Germany; u Ruprecht Haensel Laboratory, Deutsches Elektronen-Synchrotron DESY, 22607 Hamburg, Germany; v Department of Materials, ETH Zürich, 8093 Zürich, Switzerland; w Physics Department, University of Rome Tor Vergata and INFN-Sezione di Roma Tor Vergata, Via della Ricerca Scientifica 1, 00133 Roma, Italy; x Department of Molecular Sciences and Nanosystems, Ca’ Foscari University of Venice, 30172 Venice, Italy; ESRF – The European Synchrotron, France

**Keywords:** transient absorption soft X-ray spectroscopy, European XFEL

## Abstract

Femtosecond transient soft X-ray absorption spectroscopy (XAS) is a very promising technique that can be employed at X-ray free-electron lasers (FELs) to investigate out-of-equilibrium dynamics for material and energy research. Here, a dedicated setup for soft X-rays available at the Spectroscopy and Coherent Scattering (SCS) instrument at the European X-ray Free-Electron Laser (European XFEL) is presented. It consists of a beam-splitting off-axis zone plate (BOZ) used in transmission to create three copies of the incoming beam, which are used to measure the transmitted intensity through the excited and unexcited sample, as well as to monitor the incoming intensity. Since these three intensity signals are detected shot by shot and simultaneously, this setup allows normalized shot-by-shot analysis of the transmission. For photon detection, an imaging detector capable of recording up to 800 images at 4.5 MHz frame rate during the FEL burst is employed, and allows a photon-shot-noise-limited sensitivity to be approached. The setup and its capabilities are reviewed as well as the online and offline analysis tools provided to users.

## Abstract 2

A beam-splitting off-axis zone plate setup to measure transient X-ray absorption spectroscopy is presented, as implemented at the Spectroscopy and Coherent Scattering instrument at the European X-ray Free-Electron Laser.

## 1. Introduction

X-ray absorption spectroscopy is one of the most widely used techniques at synchrotron radiation facilities around the world for investigating the local structure and electronic properties of atoms in solids and molecules on surfaces or in solutions (Bianconi & Marcelli, 1992 ▸; Stöhr, 1992 ▸; Bokhoven & Lamberti, 2016 ▸). Its implementation at free-electron lasers (FELs) opens the possibility of performing high-resolution spectroscopy like at synchrotrons with the added advantage of accessing ultrafast dynamics on the femtosecond timescale. Transient X-ray absorption spectroscopy (XAS) allows, for example, monitoring electron–hole dynamics (Boeglin *et al.*, 2010 ▸; Zürch *et al.*, 2017 ▸; Britz *et al.*, 2021 ▸), electron localization (Stamm *et al.*, 2007 ▸; Lojewski *et al.*, 2022 ▸), on-site Coulomb repulsion (Baykusheva *et al.*, 2022 ▸), lattice excitation (Rothenbach *et al.*, 2019 ▸, 2021 ▸), magnetic order (Agarwal, 2022 ▸) and ultrafast phase transitions (Cavalleri *et al.*, 2005 ▸). Monochromatic soft X-ray pulses of a few nJ of energy are easily delivered by FELs and contain a few 10^7^ photons, which is several orders of magnitude more than the intensity delivered by synchrotron-based femto-slicing facilities (Holldack *et al.*, 2014 ▸). At the photon shot-noise limit, a single-shot signal-to-noise ratio (SNR) at the level of a few thousands is thus achievable. However, the X-ray pulses generated by self-amplified spontaneous emission (SASE) at a FEL feature very high pulse-to-pulse intensity fluctuations after a monochromator (Saldin *et al.*, 1998 ▸). It is therefore essential to normalize the transmitted signal by measuring the incoming radiation intensity before the sample (*I*_0_). The main challenge in measuring femtosecond XAS in the soft X-ray regime, where small changes in the spectra have to be detected, is precisely this normalization scheme.

XAS at FELs was pioneered by Bernstein *et al.* (2009 ▸), where the *I*_0_ normalization was achieved by using a half of a sample such that one half of the X-ray beam was propagating through the sample while the other half was propagating freely. A Ce-doped yttrium aluminium garnet (Ce:YAG) scintillator screen placed in front of an intensified charge-coupled-device camera (ICCD) was used for detection. This approach relies on spatial beam coherence and pointing stability of the FEL beam. An alternative approach consists of using a transmission grating to create copies of the incoming beam with the different diffraction orders and using the +1st grating order to measure the sample transmission and the −1st grating order to measure *I*_0_ (Katayama *et al.*, 2013 ▸, 2016 ▸; Brenner *et al.*, 2019 ▸; Engel *et al.*, 2020 ▸, 2021 ▸). The advantage here is that the beam intensities are linked by the grating element. Later, the sensitivity of this method was improved by combining the transmission-grating approach with a focusing zone plate component. As the beams propagate towards the detector, they are focused in front of the sample and then expand and illuminate many more pixels, thereby increasing the maximum number of photons that can be detected without saturating the detector (Schlotter *et al.*, 2020 ▸). In our improved scheme, we use an off-axis zone plate (Buzzi *et al.*, 2017 ▸; Jal *et al.*, 2019 ▸; Rösner *et al.*, 2020 ▸) which gives the possibility of separating the different zone plate orders on the detector, as we discuss below.

In this article, we review the scheme as implemented at the Spectroscopy and Coherent Scattering (SCS) instrument at the European XFEL. In Section 2, the setup is described. First, an overview of the setup and its capabilities is given in Section 2.1, followed by the design choices, specifications and fabrication details of the employed diffractive optics in Section 2.2. In Section 2.3, we review the different control aspects necessary to collect data efficiently during an experiment. Finally, in Section 2.4, we detail the beam propagation calculator that we provide to users in order to design their samples to be compatible with this setup. In Section 3, we detail the data analysis steps required to make the best use of the collected data. In Section 3.1, we introduce basic statistical concepts. In Section 3.2, we discuss the imaged beam on the detector, which leads to the flat-field correction in Section 3.3. In Section 3.4, we describe the non-linear correction and how it is calculated and applied to the data. In Section 3.5, we discuss how close these different corrections bring us to the photon shot-noise limit. In Section 3.6, we describe the offline analysis procedure available to the users and, in Section 3.7, we describe the tools we provide for the analysis during the experiment as the data are being collected. In Section 4, we showcase some examples of experiment results, starting, in Section 4.1, with the transient XAS in NiO and the impacts of the different corrections on the data. Finally, in Section 4.2, we discuss the sensitivity limits of the current setup in terms of X-ray fluence (Section 4.2.1), repetition rate (Section 4.2.2) and typical sample systems that can and cannot be measured currently with this setup (Section 4.2.3).

## 2. Setup

### 2.1. Overview

**Figure 1:**
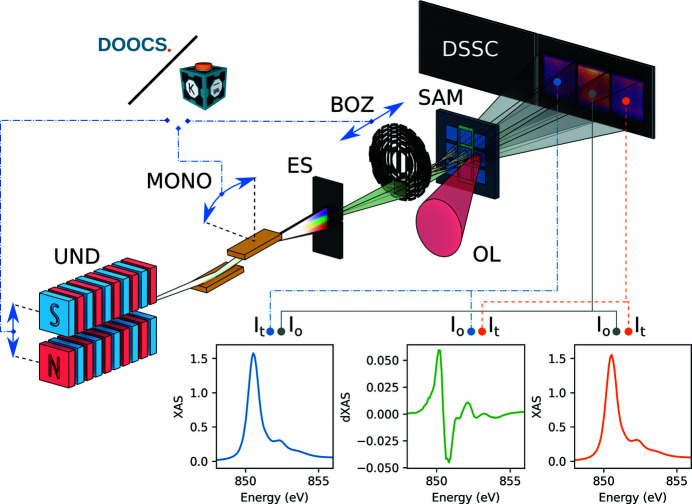
Scheme of the beam-splitting off-axis zone plate (BOZ) setup which is described in detail in the main text.

The setup implemented at the SCS instrument at the European XFEL is schematically shown in Fig. 1 ▸. The X-ray pulses are generated in the SASE3 undulator system (UND). The X-ray photon energy is determined by the fixed electron bunch acceleration energy and the variable undulator gap. The X-rays are then monochromated with the help of a variable-line-spacing grating (MONO) combined with an exit slit (ES) (Gerasimova *et al.*, 2022 ▸). The monochromatic X-ray pulses propagate through the beam-splitting off-axis zone plate (BOZ) optics. It consists of a transmission grating and a focusing zone plate in a single element. The grating splits the initial beam into three beams of approximately equal intensity. The zone plate focuses these beams shortly before the sample (SAM) and the resulting X-ray spot size on the membrane is typically 50 µm × 50 µm. The transmitted beams further expand downstream and are detected on a single monolithic sensor of the DSSC^1^ detector placed at 5.4 m. The sensor is 3 cm high and 6.2 cm wide and populated by 128 by 256 pixels. The high sensitivity of this measurement scheme is assured by using a low-noise detector and illuminating many pixels to achieve a high signal-to-noise ratio. The method’s sensitivity is then mainly limited by the number of photons detected, as we show below. The DSSC can record up to 800 frames at 4.5 MHz during the FEL train, meaning an effective repetition rate of 8 kHz. The sample consists of an array of X-ray transparent membranes, with each of the three X-ray beams passing through a separate membrane window. The middle membrane window consists of the bare substrate, while the right and left membranes each consist of the thin film under investigation. To record a spectrum, the X-ray photon energy can be scanned by varying together the undulator gap, the monochromator energy and the BOZ position along the X-ray beam, with the help of the Distributed Object Oriented Control System (DOOCS) (Grygiel *et al.*, 1996 ▸) and the Karabo control system (Hauf *et al.*, 2019 ▸), as shown in Fig. 1 ▸ by dash-dotted lines connecting blue double-headed arrows. Finally, for stroboscopic or single-shot pump–probe experiments, an optical pump laser (OL) can be focused onto one of the membranes to excite it (Pergament *et al.*, 2016 ▸). The time delay between the optical pump and X-ray probe pulses can be controlled by an optical delay line (not shown in Fig. 1 ▸ for simplicity). By defining regions of interest, the transmitted intensity of each of the three separated beams can be computed from the DSSC detector images. The XAS of the unexcited sample can be determined from the intensity of the beam going through the bare membrane (grating 0th order) and the beam passing through the unexcited sample (grating +1st order), as shown by the blue curve in the bottom-left plot in Fig. 1 ▸. Similarly, the XAS of the excited sample can be determined simultaneously, as shown by the orange curve in the bottom-right plot in Fig. 1 ▸. Finally, the pump-induced XAS change can be determined as well simultaneously from the intensity of the beam going through the unexcited sample and the beam going through the excited sample, as shown by the green curve in the bottom-center plot in Fig. 1 ▸. In the next sections, we present the design and fabrication of the BOZ optics, the different control aspects necessary to collect a spectrum, and, finally, the tools available to users to aid in the design of samples for this setup.

### 2.2. Diffractive optics

Horizontally, the grating structure of the BOZ optics splits the beam into different orders. The period of the grating structure is chosen to provide an angular separation of the diffraction orders of 3.1 mrad, which is just sufficient to prevent the beams from overlapping as they propagate 5.4 m up to the DSSC detector placed at the end of the SCS experiment hutch. This gives, for example, a grating structure period of 465 nm to operate around the Ni *L*_3,2_ edges at 860 eV.

To detect as many photons as possible without saturating the detector, the beams have to be as large as feasible. This means that the focal length of the Fresnel zone plate component of the BOZ optics should be chosen as small as possible. For this setup, we have chosen a focal length of 250 mm to ensure that the sample can be placed just upstream of the zone-plate focus in its most upstream position. Then, by using the 190 mm scanning range of the sample along the beam propagation, we can control the spot size of the X-rays on the sample, from tight focus at the zone plate focus to much larger beams in the most downstream position. Similarly to the grating structure, the Fresnel zone plate structure creates diffraction orders. Considering only the lowest diffraction orders, we have the +1st zone plate order, which is focused downstream at the zone plate focus, the 0th zone plate order, which is unfocused and simply propagates through, and the −1st zone plate order, which is diverging. With an on-axis zone plate, all these different orders would spatially overlap on the detector (Schlotter *et al.*, 2020 ▸). Here, we use an off-axis part of the zone plate, as schematically shown in Fig. 1 ▸ with the beam hitting the bottom instead of the center of the zone plate, to vertically separate the different orders on the detector. In our setup, this off-axis component, measured as the distance between the BOZ optics center and the optical axis for the zone plate, is chosen to be 0.55 mm. For the Ni *L*_3,2_ edges at 860 eV, this gives an off-axis Fresnel zone plate structure with an outermost zone width of 179 nm for a zone plate aperture of 0.8 mm × 0.8 mm.

The intensity ratio of the three focused beams can be controlled by two different design parameters in the BOZ pattern as described in the pattern-inversion method and the pattern-shift method (Döring *et al.*, 2020 ▸). We applied the latter method with a shift parameter of *s* = 0.32, as it gives higher overall efficiency.

The BOZ elements were made from single-crystal silicon membranes (Döring *et al.*, 2020 ▸). The 1 µm-thick silicon membranes (Norcada Inc., Edmonton, Canada) were sputter-coated with a 10 nm chromium layer and then spin-coated with a 70 nm polymethylmethacrylate (PMMA) resist. After electron-beam lithography (Vistec EBPG5000+, operated at 100 keV electron energy) of the BOZ patterns and subsequent development, the resist patterns were transferred into the Cr mask by reactive ion etching in a Cl_2_/O_2_ plasma. After removal of the PMMA resist in acetone, the pattern was etched down to a depth of about 700 nm into the silicon membranes by reactive ion etching in an SF_6_/C_4_F_8_/O_2_ plasma. Finally, the Cr mask layer was removed to yield pure silicon structures.

### 2.3. Controls

To record an XAS spectrum, the monochromator (Gerasimova *et al.*, 2022 ▸) is scanned continuously back and forth between two energy endpoints. The 120 m-long undulator system is controlled through the DOOCS control system (Karabekyan *et al.*, 2012 ▸, 2013 ▸). A DOOCS middlelayer (ML) server provides an interface to specify the undulator photon energy and, in turn, controls the gap size of each undulator. During a scan, the Karabo control system ensures that the undulator photon energy follows the monochromator photon energy through a feedback loop that interfaces the DOOCS ML server through Karabo–DOOCS bridging software. This combination allows spectra covering tens of eV to be recorded.

However, to maintain full lasing of the undulators, the relative variation in the undulator deflection parameter Δ*K*/*K* and, respectively, the relative change in the magnetic field strength Δ*B*/*B* for the undulator system should not exceed the Pierce or FEL parameter ρ (Pierce, 1950 ▸; Bonifacio *et al.*, 1984 ▸; McNeil & Thompson, 2010 ▸). In the case of the SASE3 undulator system, this relative change of the deflecting parameter Δ*K*/*K* should not exceed the value of 1 × 10^−3^. The magnitude of the magnetic field depends on the undulator gap *g* and the undulator period λ_u_ and is described by an exponential decay according to the expression

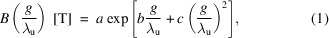

where, in the case of the U68 undulator with period λ_u_ = 68 mm, the parameters have the values *a* = 3.214, *b* = −4.623 and *c* = 0.925. By applying the partial differential method with respect to changes of the gap Δ*g*, the boundary condition for full lasing can be determined,

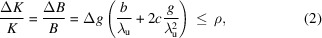

giving

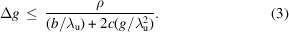

Assuming that the working range of the U68 undulator gap is 10–25 mm, the maximum deviation of the gap between undulators of one system should not exceed Δ*g* = 15–17 µm to maintain the full lasing condition.

If the undulator system is set to follow the monochromator with a scanning speed of 1 eV s^−1^, we need to determine the undulator gap scanning speed. From the undulator resonance equation for the first harmonic at small observation angles, 

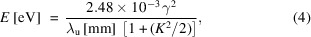

where γ is the relativistic Lorentz factor for the electron, we can use the the partial differential method with respect to the undulator gap change. The resulting gap velocity values for 10 mm and 25 mm undulator gap are 10 µm s^−1^ and 2 µm s^−1^, respectively. Measurements made on a system of four undulators showed that, even without forced synchronization of the axes of the undulators, the maximum deviation for a gap velocity of 0.856 mm s^−1^ corresponds to 40 µm (Karabekyan *et al.*, 2013 ▸). It was also shown that this dependence is close to linear. By linear approximation for a gap velocity of 10 µm s^−1^, it could be concluded that the maximum deviation of the gap of undulators in one system would not exceed 0.5 µm. This value confirms that, by coupling the monochromator axis and the gap axes of the undulator system, the full on-the-fly lasing condition for soft X-ray beamlines can be achieved, even with a few seconds delay in communication between the undulator system and the monochromator.

One drawback of employing diffractive optics is that their properties are wavelength-dependent. For the BOZ, this means that both the focal distance and the grating diffraction angle are proportional to the photon energy. During an extended energy scan, both the X-ray spot size and the beam pointing on the sample can vary significantly. To compensate for these effects, we use a three-axis linear piezo-motor stage to displace the BOZ along the X-ray beam to a position calculated from the monochromator readback energy. This ensures that the X-ray spot size and position on the sample remain constant during the energy scan. The change in BOZ position along the X-ray beam Δ*z* due to a change in photon energy Δ*E* can be calculated from the zone plate focal length *f* at the design energy *E*_0_ using 

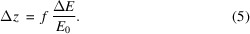

As an example, considering an energy scan spanning both Ni *L*_3,2_ edges from 845 eV to 875 eV for a zone plate with a design energy of *E*_0_ = 860 eV with a focal length of *f* = 230 mm, the change in BOZ position along the X-ray beam Δ*z* is 8 mm.

### 2.4. Beam propagation

In contrast to the setup of Schlotter *et al.* (2020 ▸), where independent manipulators were used to align individual sample and reference membranes in the beam, the setup at the SCS instrument has only one sample manipulator. Therefore, the sample has to be precisely designed to fit with the three-beam geometry from the start. To facilitate this, we publicly provide the *BOZcalc* Python package (SCS, 2022*a* ▸) to calculate the beam propagation and display projections at the sample and detector plane.

**Figure 2:**
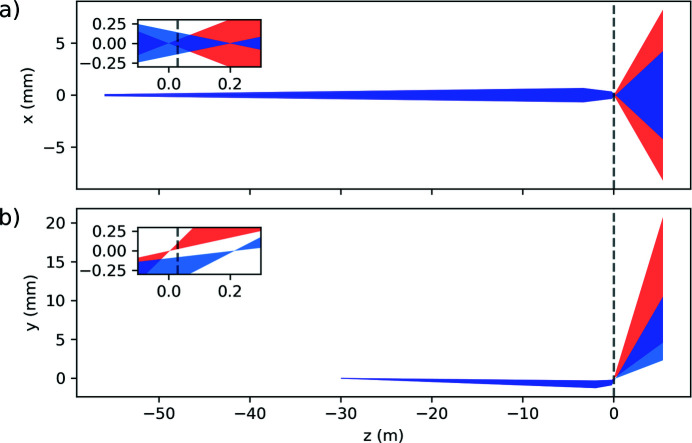
(*a*) Horizontal and (*b*) vertical beam propagation from the intermediate source point to the detector. The position of the sample is shown as a dashed vertical line. The inset in each figure shows a zoomed-in region around the sample position. The red-filled areas represent the 707 eV photon beams from the fundamental harmonic of the undulators. The blue-filled areas represent the beams at 1414 eV photon energy originating from the second harmonic of the undulators. The vertical separation between fundamental and second-harmonic beams near the zone-plate focus arising from the off-axis component of the diffractive optics can be used to block the unwanted radiation.

For example, in Fig. 2 ▸, the horizontal and vertical beam profiles as a function of the distance from the interaction point, starting from the horizontal and vertical intermediate source points up to the DSSC detector, are displayed. From the intermediate source point, the X-ray beam propagates down to the SCS instrument and is slightly focused by the Kirkpatrick–Baez mirrors (Mercurio *et al.*, 2022 ▸) up to the BOZ. After that, the beam is strongly focused and then expands until it reaches the DSSC detector. The advantage of using a zone plate with an off-axis component becomes evident in Fig. 2(*b*) ▸, where the undulators’ fundamental (red) and the second harmonic (blue) are spatially separated. This allows the sample to be used as a harmonic sorting aperture, blocking the unwanted undulator second-harmonic contribution that is not always suppressed by the beamline offset mirrors.

**Figure 3:**
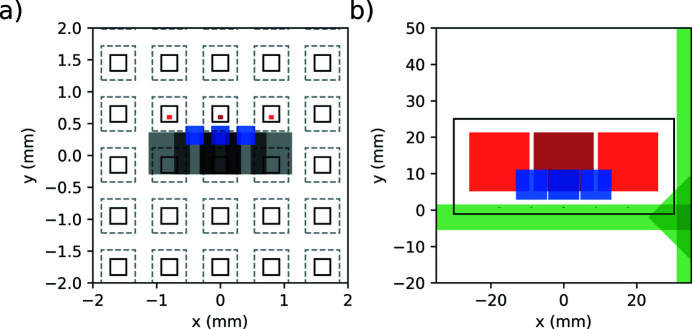
BOZ calculator showing the beams at (*a*) the sample and (*b*) the detector planes. The focused 1st zone-plate order beam footprint at the respective positions is shown as red for the fundamental and blue for the second harmonic. The 0th zone plate order is shown as gray. Individual membrane windows and the etching lines are shown in (*a*) as continuous and dashed lines, respectively. In (*b*) the DSSC sensor is represented by the black rectangle and the DSSC filter mount is represented by the green shapes.

In Figs. 3 ▸(*a*) and 3(*b*), the results of a calculation, using the *BOZcalc* Python package, of the three beams’ positions and shapes at the sample plane, respectively, at the detector plane are shown. All the calculations are performed within a *Jupyter* notebook. In the sample plane in Fig. 3 ▸(*a*), a membrane array is displayed as a series of black rectangles. The etching facets on the back of the substrate are represented as gray dashed lines. The red squares indicate the expected beam size for the focused 1st order beam of the zone plate at the given sample position. The overlapping gray squares represent the un­focused 0th order of the zone plate. The blue squares are the expected beam position and size of the second-harmonic beam from the undulator. In this arrangement, the unwanted second-harmonic radiation would be blocked by the sample frame, while the fundamental beam would propagate through the sample membranes. In the detector plane shown in Fig. 3 ▸(*b*), the black rectangle represents the DSSC module used to record the beams’ intensity, while the green shapes represent the DSSC filter mount that is opaque to X-rays. The red squares show how the three beams expand before reaching the DSSC to fill the sensor area after being focused by the zone plate in front of the sample. It is worth noting that the undulators’ second harmonics would overlap with the other beams of interest if they were not blocked by the sample. It is also worth mentioning that the grating 2nd order, zone plate 2nd order of the second undulator harmonic, will exactly overlap with the grating 1st order, zone plate 1st order of the undulator fundamental, and therefore cannot be separated with an aperture. However, the intensity of the 2nd grating order is determined by the duty cycle of the grating structure and is exactly zero for an even duty cycle. As a consequence, the pattern shift method shows the important advantage of suppressing a potential contamination of the signal by the second-harmonic beams appearing in the 2nd grating order (Döring *et al.*, 2020 ▸). The unfocused beams from the 0th zone plate order are the barely visible small spots seen on the filter mount near the bottom of the DSSC module. It is also important to block these beams as they would otherwise saturate the illuminated pixels. The input controls of the *BOZcalc* calculator can be interactively adjusted. This allows, for example, to quickly check the expected beam size at different sample distance.

Having presented the setup and the tools available to users to design samples compatible with this setup, we now detail the data processing and analysis required.

## 3. Data processing

### 3.1. Statistics

The absorption of light propagating through a sample with thickness *d* is given by the Beer–Lambert law,

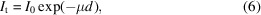

where *I*_0_ is the incoming photon intensity, *I*_t_ is the transmitted photon intensity after the sample, μ is the inverse of the absorption length, and *d* is the sample thickness. The sample transmission is *T* = *I*_t_/*I*_0_, and the X-ray absorption *A* is 

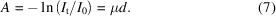

The other important quantity to determine is the SNR of the measurement, which is given by propagating uncertainties (Meija & Mester, 2007 ▸; Schlotter *et al.*, 2020 ▸), 

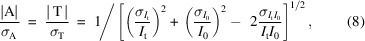

where |…| is the absolute value, σ is the standard deviation, and 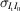 is the covariance. This last term is essential as it reduces drastically the variance of the measured ratio, as both *I*_t_ and *I*_0_ measurements fluctuate mainly due to a common cause which is the large intensity fluctuation of the monochromitized SASE pulses.

To determine the photon shot-noise limit, we consider the ideal case where the incoming intensity is constant, and the only source of noise comes from measuring the *I*_t_ and *I*_0_ intensities in the detector. This leads to 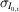 = 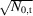 , where *N*_0,t_ is the number of photons. In addition, there is no longer any correlation between the two intensity measurements such that 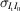 = 0. Simplifying equation (8) in the photon shot-noise limit gives 

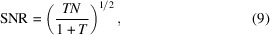

where *N* is the number of photons in the *I*_0_ beam and *T* is the average transmission. For a weakly absorbing sample with a transmission close to 1, the SNR limit is thus 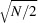 , while for a strongly absorbing sample, *i.e.* with a transmission of 0.1, the SNR limit will be dominated by the noise in the transmitted beam with fewer photons, giving a reduced SNR limit of 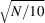 in this example.

When data are recorded, several shots are taken at the same photon energies (or time delay) and have to be averaged together. The first approach is to compute the transmission *T*_*i*_ for each of the *M* shots and average them together, such that *T* = 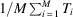 . The uncertainty is simply given by the standard deviation σ_*T*_ of *T*_*i*_ and the single-shot SNR is obtained from the left-hand side of equation (8),

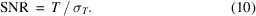

However, shots with weak intensity, due to the high fluctuations introduced by monochromatizing the SASE pulses, tend to be noisier and dominate the uncertainty in the measurement. A better approach is to first compute the summed incoming 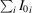 and transmitted 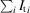 intensities before calculating the averaged transmission *T*_w_, 

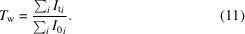

Here, we are simply summing up the photons detected over many shots together. This is identical to computing a weighted average of *T*_*i*_ with *I*_0*i*_ as weight, *i.e.* intense shots should contribute more and weak shots should contribute less to the mean. The uncertainty in the measurement is now given by the weighted standard deviation, 

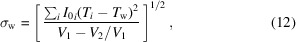

with 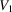 = 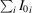 and 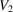 = 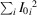 . The single-shot weighted SNR is then 

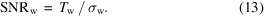




### 3.2. Imaging

**Figure 4:**
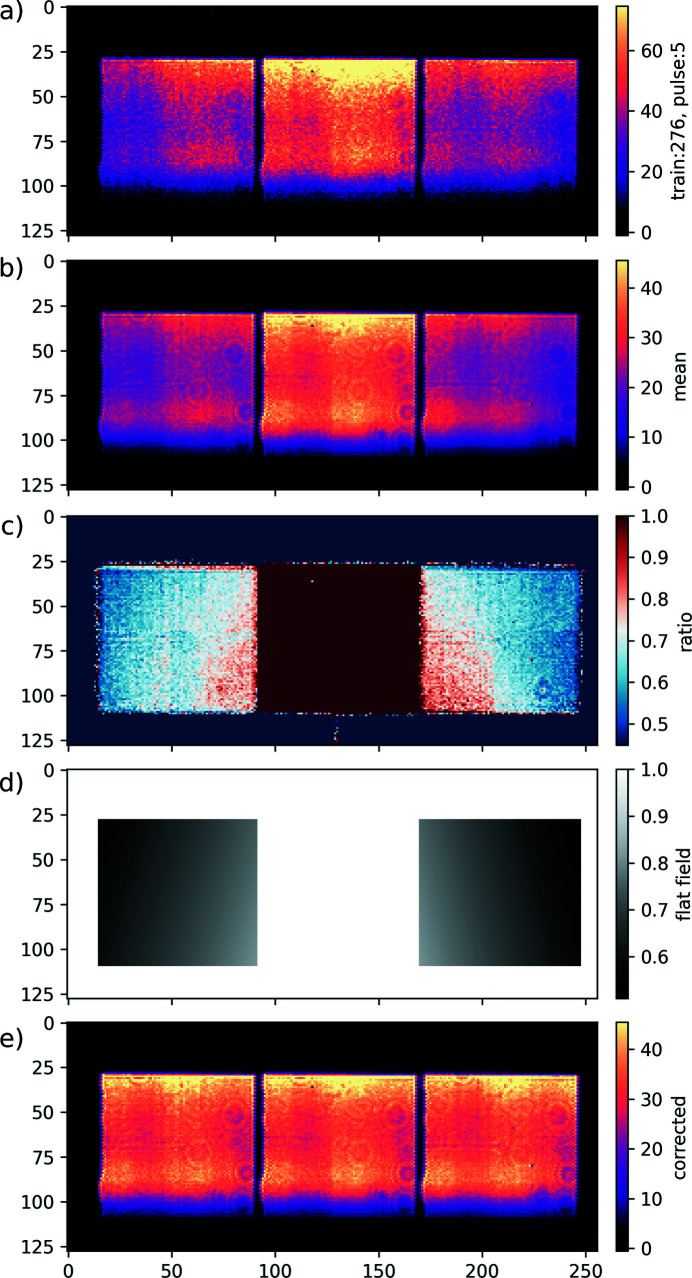
(*a*) Dark-corrected single-pulse image of the DSSC sensor showing the three imaged beams (−1st, 0th and +1st order). (*b*) Dark-corrected average image of a single DSSC sensor showing the three imaged beams based on a 5 min-long data acquisition recording 3000 FEL trains with 15 X-ray pulses per train. (*c*) Image showing each beam normalized by the 0th order beam. (*d*) Fitted flat-field correction. (*e*) Dark-corrected and flat-field-corrected average image in a single DSSC sensor. The three imaged beams now appear as identical copies of the initial beam.

To characterize the sensitivity of the setup, we collect a set of data without samples in the beam in which case the transmission is known to be exactly *T* = 1. In Fig. 4 ▸(*a*), a single SASE pulse is imaged on the DSSC sensor, showing the three characteristic beams from the diffractive optics. To simplify the discussion in the rest of the manuscript, we identify these three beams by their −1st, 0th and +1st grating order alone, omitting to mention their +1st zone-plate order part. The shape of each beam is roughly a square and is determined by the aperture size of the zone-plate optics. A four-slit system directly upstream of the diffractive optics is used to control the incoming beam size and to ensure separation of the diffracted beams on the DSSC detector. The average dark-corrected image over a 5 min-long dataset consisting of 3000 FEL trains with 15 X-ray pulses per train is shown in Fig. 4 ▸(*b*). The first point to note is that both the −1st and the +1st grating orders are somewhat weaker than the 0th grating order in the middle. This is due to the design of the diffractive optics and the resulting diffraction efficiency in each order, which can be tuned (Döring *et al.*, 2020 ▸). The second point to note is that the beam intensity of the right beam decreases on its right side and, similarly, the left beam intensity decreases on its left side. We suspect this effect to be an intrinsic property of the diffractive optics and not a fabrication issue, as it occurs for all the optics we tested. The variation of intensity across different beams makes the measured transmission spatially dependent and deviating from 1.0. In the next section, we detail how this effect can be corrected.

### 3.3. Flat-field correction

The ratios between each beam and the 0th grating order beam are shown in Fig. 4 ▸(*c*). One notices the presence of gradients in the −1st/0th and the +1st/0th ratios, with values ranging from 0.55 up to 0.85. The orientation of the gradients is along the diagonal direction with respect to the square shape of the beams, and they appear to be mirror images of each other. To correct for the gradients, we fit a plane defined by *ax* + *by* + *cz* + *d* = 0, where *x* and *y* are the horizontal and vertical positions, respectively, of a pixel in the sensor and *z* is the value of the ratio computed for the corresponding pixel. We can impose a horizontal mirror symmetry such that both gradients are fitted with only four fitting parameters, *a*, *b*, *c* and *d*, in total. The result of the fitting is shown in Fig. 4 ▸(*d*), where a value of 1.0 is set everywhere, except where the −1st and +1st beams are located, in which case the fitted plane and its mirror are evaluated. Dividing the data shown in Fig. 4 ▸(*b*) by the normalization shown in Fig. 4 ▸(*d*) results in the data shown in Fig. 4 ▸(*e*). The imaged beams now appear as three identical copies of the same initial beam image, as we would expect from the property of a grating. We call this normalization step the flat-field correction.

**Figure 5:**
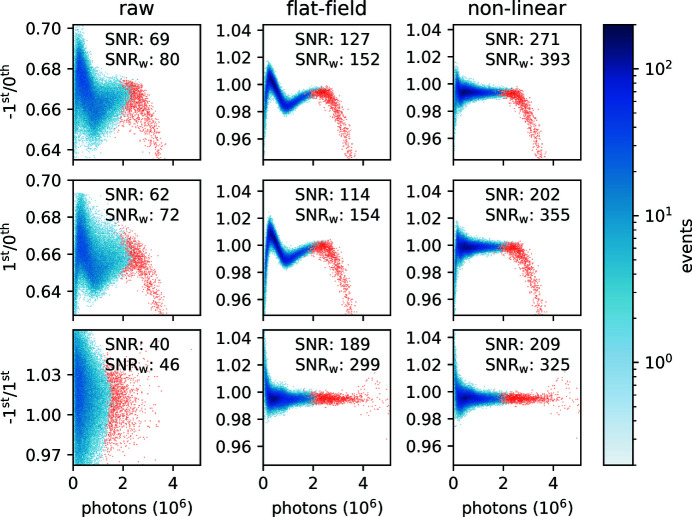
Two-dimensional histograms of the three ratios (−1st/0th grating order in the top row, 1st/0th in the middle row, and −1st/1st in the bottom row) as a function of the number of photons in the 0th order for dark-corrected data in the left ‘raw’ column, for the dark-corrected and flat-field-corrected data in the middle ‘flat-field’ column, and finally for the dark-corrected, flat-field-corrected and non-linear-corrected data in the right ‘non-linear’ column. Data that contain saturated pixels are shown as red, as opposed to blue where no saturation occurs. In each plot, the SNR and weighted SNR (SNR_w_) of the non-saturated events are indicated.

To better understand the statistical distribution of the data, we extract the intensity of the three beams by defining three regions of interest (ROIs) around each beam and summing up the measured pixel values within them after dark correction. We then compute the three possible ratios −1st/0th, +1st/0th and −1st/+1st grating orders as a function of the intensity in the 0th grating order for every pulse in every train in the dataset. The resulting two-dimensional histograms are shown in Fig. 5 ▸. The column on the left in Fig. 5 ▸ labeled ‘raw’ shows data that are only dark-corrected. We can see that they are quite dispersed. In these plots, the data in red show the pulses where at least one pixel is saturated, making them not reliable, while the data in blue show no saturation of the DSSC detector. On each plot, the SNR given by equation (10) and weighted SNR (SNR_w_) given by equation (13) are shown for the non-saturated data. After applying the flat-field correction, which compensates for some diffraction-efficiency variation in the diffractive optics, we obtain the data shown in the middle column in Fig. 5 ▸, labeled ‘flat-field’. We can see that the improvement is significant and that these first corrected data are much less dispersed. We also see a large increase in SNR_w_ by a factor of six for the −1st/+1st ratio, from 46 to 299. For the two other ratios, the improvement is more moderate and only a factor of two. In addition, the measured transmission now clearly appears non-linear as a function of the intensity in the 0th order, a feature that was in part hidden in the noise before.

In practice, the zone plate can be slightly rotated with respect to the DSSC sensor. This can be taken into account in the fitting procedure of the flat-field correction by lifting the horizontal symmetry requirement at the cost of doubling the number of fitting parameters from four to eight. In addition, a ratio is only properly defined where we have enough intensity; otherwise, it creates outliers. The process of finding ROIs encompassing bright enough pixels and excluding outliers can be time consuming. To solve this problem, a more reliable approach was derived. The idea is based on the change displayed by flat-field-corrected data as compared with the raw data and is shown in Fig. 5 ▸. If we bin the data in *k* = 40 small intervals of intensity in the 0th order, then, within each of these intervals, the spread in the data for each of the three ratios is reduced by the flat-field correction. This spread is naturally measured with the standard deviation over each *k* interval, σ_*k*_. To fit the flat-field correction, we introduce the following criterion *J*_ff_,

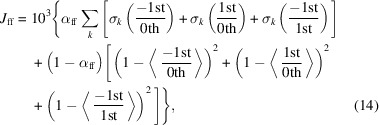

where the term in α_ff_ is the sum over all *k* intervals of the standard deviation for all three ratios. The term in (1 − α_ff_) is a regularization term to keep the mean 〈…〉 of each of the three ratios around unity. Minimizing *J*_ff_ as a function of the eight fitting parameters (four for each plane without mirror symmetry) proved to be more reliable and gave better estimates of the flat-field correction compared with fitting a plane to the ratio of the mean images of each beam. In practice, the fitting procedure converges within a few iterations and a regularization parameter α_ff_ of about 0.1 works well and is not critical. By making the *k* intervals small enough, we can be sure that the non-linear trend, visible in Fig. 5 ▸ for the flat-field-corrected data, is not contributing to *J*_ff_. While the improvement brought in by the flat-field correction in the data is very significant, it is clear that the non-linearity observed in Fig. 5 ▸ needs to be addressed if we want to make the best use of the available data.

### 3.4. Non-linear correction

The mini-silicon drift detector (MiniSDD) camera is a linear system with an analog chain linearity error better than 0.25% (Grande *et al.*, 2019 ▸) and an analog-to-digital converter (ADC) with integral non-linearity (INL) and differential non-linearity (DNL) better than 0.5 least significant bits (LSB) and 0.32 LSB on the full range, respectively (Hansen *et al.*, 2013 ▸). Nevertheless, the remaining non-linearity that we observe needs to be addressed to further improve the data quality. Here, we assume as a first approximation that the DSSC single-pixel response is non-linear as a function of the incoming photon intensity. Moreover, we assume that this non-linearity can be corrected by a *pixel-independent* non-linear correction function *F*_nl_ that only deviates slightly from the ideal linear detector response. We model *F*_nl_(*x*) over the integer range from 0 to 511, representing the 9 bits of the DSSC pixel output values, as a piecewise constant function composed of *S* segments. It starts from a user-defined low level *L* and goes up to a high level *H*. In practice, we have *S* = 80, *L* = 40, which is below the dark pedestal, and *H* = 511. To apply this correction function to the collected raw data output of the DSSC, we proceed with the following algorithm:

(1) Replace integer value *x* with float value *F*_nl_(*x*) in both dark run data and run data.

(2) Subtract from the run data the pulse-resolved mean dark value.

(3) Divide run data by flat-field normalization.

(4) Sum run data pixel values over each ROI.

**Figure 6:**
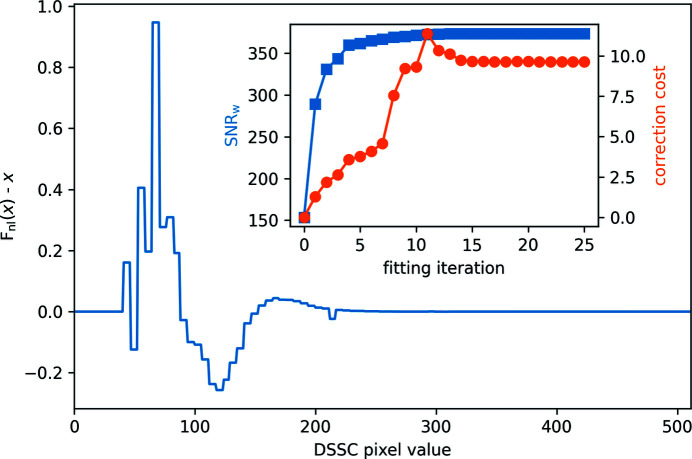
Fitted non-linear deviation from the ideal detector response *F*_nl_(*x*) − *x* as a function of the DSSC pixel values. Inset: the evolution of the weighted SNR (blue squares) and the correction cost (orange circles) as a function of the fitting iteration number.

We then compute the weighted variance 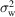 according to equation (12) for each of the three intensity ratios measured in each ROI. The goal is then to fit the *pixel-independent* non-linear correction function *F*_nl_ in order to maximize the SNR_w_ of the −1st/0th and the +1st/0th ratios. For this, we calculate the following criterion *J*_nl_, 

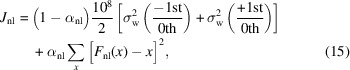

with α_nl_ being a user-defined parameter between 0 and 1 controlling the strength of the regularization term. This term prevents the fitting from diverging to an unrealistic non-linear correction function by keeping the correction cost, *i.e.* the deviation from the ideal detector response, as small as possible. In practice, we use α_nl_ = 0.5 as a default value. We then minimize *J*_nl_ as a function of the *S* piecewise constant values modeling *F*_nl_. This computation typically takes 2 to 8 h on a single node on the Maxwell computational resources operated at DESY and accessible to the users of the European XFEL. In Fig. 6 ▸, the fitted non-linear correction deviation *F*_nl_(*x*) − *x* is shown and is indeed small. Here, a maximum deviation of less than 1 for an input value of about 80 is seen. As the DSSC was operated at a frame rate of 4.5 MHz, the actual input value recorded in this dataset does not extend beyond 280 (Porro *et al.*, 2021 ▸). This explains why the deviation is zero in the range 280 to 511. The inset in Fig. 6 ▸ shows the evolution of each component of the minimization criterion *J*_nl_ as a function of the fitting iteration number, with SNR_w_ being 1/[10^−8^ *J*_nl_(α_nl_ = 0)]^1/2^ and the correction cost being *J*_nl_(α_nl_ = 1). One can see that, within a few iterations, SNR_w_ increases significantly for a very moderate increase in the correction cost. With further iterations, SNR_w_ increases slightly to a plateau, at the cost of a much larger increase in the correction cost. At this stage, the only gain in minimizing *J*_nl_ is by reducing the correction cost, as shown by the small reduction around iteration 15 while SNR_w_ remains constant. Overall, within 25 iterations, the fitting has converged. In Fig. 5 ▸, the data corrected for dark, flat-field and non-linear response are shown in the right column labeled ‘non-linear’. It is evident from these plots that the data are now much more linear, with the exception of the saturated data in red, which are discarded from further analysis in any case. We see an increase by a factor of 2.5 in SNR_w_ with the addition of the non-linear correction for the −1st/0th and the +1st/0th ratios. For the −1st/+1st ratio, the gain is much smaller. This is easy to understand, considering that, since the +1st and −1st are very similar in intensity, their ratio is largely independent of detector non-linearity. This, in turn, motivates the omission of the −1st/+1st ratio in equation (15). Interestingly, we note that discarding the saturated pulses when computing the −1st/+1st ratio might not always be the best strategy, as these data do not apparently deviate significantly from the non-saturated data. This could be because the increase in beam intensity is well determined by the many non-saturated pixels and not dominated by a few saturated pixels in each beam, in contrast to beams of dissimilar intensity, where saturation occurs only in one of the beams. In summary, combining a flat-field and a non-linear correction that can be efficiently calculated, we significantly improved the collected data. In the next section, we discuss how close the corrected data are to the photon shot-noise limit.

### 3.5. Photon shot-noise limit

**Figure 7:**
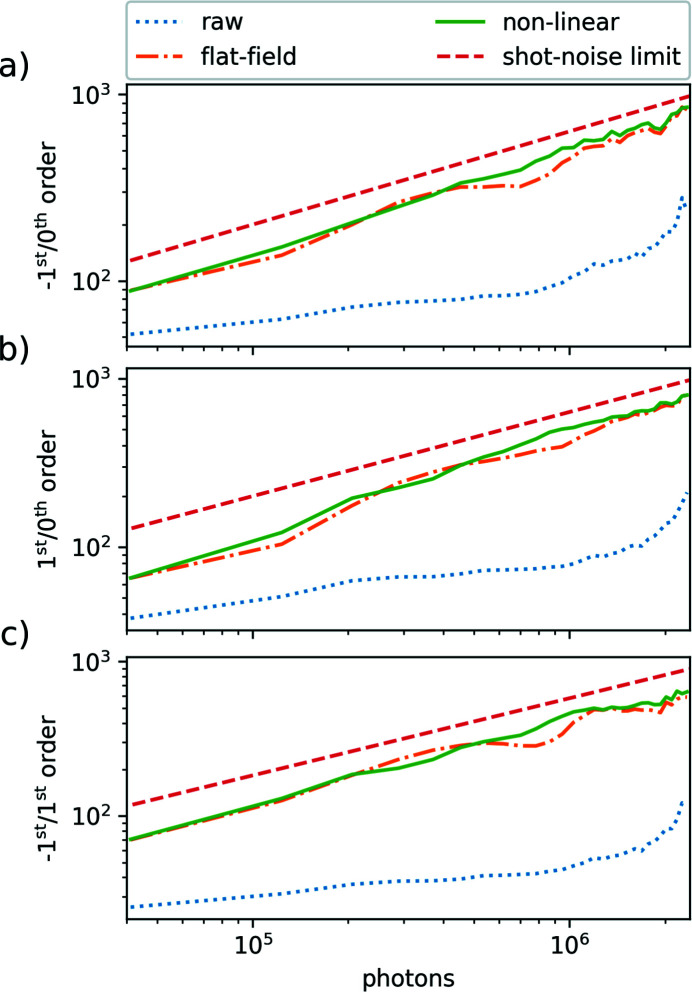
SNR (inverse of the standard deviation) of the data binned as a function of the intensity in the 0th order for (*a*) the −1st/0th order, (*b*) the +1st/0th order and (*c*) the −1st/+1st order. Data that are only dark-corrected are shown as dotted blue lines. Data that are also flat-field corrected are shown as dot-dashed orange lines. Data that are also corrected for non-linearity are shown as continuous green lines. The photon shot-noise limit is shown as dashed red lines.

To address the question of how close the corrected data are to the photon shot-noise limit, we plot in Fig. 7 ▸ the inverse of the standard deviation of the data binned as a function of the intensity in the 0th order. The data, which are only dark-corrected, are shown as blue dotted lines. Data that are additionally flat-field corrected are shown as orange dash-dotted lines. Data that are also non-linear corrected are shown as continuous green lines. The photon shot-noise limit given by equation (9) is shown as red dashed lines in Fig. 7 ▸. With the flat-field correction, data are already approaching the photon shot-noise limit closely. The effect of correcting for non-linearity is not visible in this plot as we only show the inverse of the standard deviation of the binned data and not the systematic deviation from 1. From Fig. 7 ▸, we conclude that we are making efficient use of every photon detected by the DSSC detector, using the detailed data correction steps.

Before looking at actual time-resolved transient XAS measurements on the sample and confirming that the data treatment gives sensible results, we discuss the different corrections we apply to the data and their origin. At the moment, we lack a predictive model for the position-dependent diffraction efficiency of the zone plate, which we correct with the flat-field correction. Nevertheless, it seems that the plane approximation to that unknown dependence is sufficient. For the non-linear correction, the MiniSDD DSSC pixel response is linear within the expected margins (Grande *et al.*, 2019 ▸; Hansen *et al.*, 2013 ▸; Porro *et al.*, 2021 ▸), but, as we have shown, the data quality can be further improved by correcting the remaining non-linear behavior with a *pixel-independent* non-linear correction function.

Finally, we point out that taking the necessary calibration data, consisting of a dark run of 1 min and a run without sample for a few minutes, is very quick to acquire. Moreover, the computed correction should remain valid unless the X-rays or the BOZ are realigned due to a beam pointing drift, in which case a new set of calibration data needs to be taken. Fortunately, the data processing that we detailed allows us to completely mitigate these effects and to reach the desired regime, where the sensitivity of the setup is only limited by the number of detected photons.

### 3.6. Offline analysis

The analysis procedure, detailed in the previous section, is made publicly available to all users as routines in the *SCS toolbox* Python package (SCS, 2022*c* ▸), with example *Jupyter* notebooks readily available in the online documentation (SCS, 2022*b* ▸). The workflow is quite simple and detailed here. First, we use a dedicated notebook to calculate the flat-field and non-linear correction on a set of data recorded without a sample. This computation takes several hours for the non-linearity correction but only a few tens of minutes for the flat-field correction. The result is saved in a small JavaScript Object Notation (JSON) file that can be used later on to process data, both for the offline analysis and for the online analysis. To speed up the data analysis during the beam time, an intermediate JSON correction file is saved as soon as the flat-field correction is finished. Second, the processing of data recorded with a sample is split into two parts, each having its dedicated notebook. The first part consists of processing the DSSC data, applying all the detailed corrections and computing the intensity in each beam, and finally saving the result in an intermediate small data file. The second part is to load one or several of these small data files and to compute the XAS spectra or time delay traces with a binning procedure. This part can be easily modified and adapted by the users to their needs during and after the beam time.

Offline analysis programs and notebooks make use of European XFEL’s *Extra-data* package (Fangohr *et al.*, 2018 ▸), which provides convenient access to the data files written at the European XFEL. The Python-based *Extra-data* framework (Extra-data, 2022 ▸) makes data available through common data science tools and objects such as NumPy’s arrays (Harris *et al.*, 2020 ▸), xarray (Hoyer & Hamman, 2017 ▸) and dask array (Dask, 2016 ▸). In particular, it is thanks to the multiprocessing capability offered by dask array that the computation time of the non-linearity correction could be reduced from days to just a few hours.

*Jupyter* notebooks are used by European XFEL users and staff to explore and analyze experiment data (Fangohr *et al.*, 2020 ▸). The *JupyterHub* installation of the Maxwell cluster provides remote execution of *Jupyter* notebooks using the Maxwell resources and thus provides an alternative to remote X, FastX or other remote-access technologies. This is of particular value, as the datasets recorded at the European XFEL can be so large — up to a petabyte for a five-day-long beam time recording the full DSSC detector with 800 frames per train — that they typically stay at the facility and need to be analyzed remotely after the beam time. Here, for experiments employing a single DSSC module and recording a few tens of pulses per train, the amount of data generated is more moderate, of the order of a few tens of terabytes per beam time. The use of *Jupyter* notebooks can also help to make data analysis and publications more reproducible (Beg *et al.*, 2021 ▸).

### 3.7. Online analysis

FEL beam times are both expensive and limited. It is therefore crucial for users to be able to analyze the data in real time in order to steer the experiment and maximize the scientific output. Karabo is designed to support concurrent initial analysis during data acquisition (Hauf *et al.*, 2019 ▸; Fangohr *et al.*, 2018 ▸). It is a distributed software that consists of small pluggable components, so-called devices, that represent various components: a detector, a piece of equipment such as a sensor, or a control and analysis procedure such as a scanning routine. Karabo also includes a graphical user interface that allows feedback on the control system.

There are different possibilities to achieve real-time data analysis at the European XFEL, which is developing a related Karabo device that describes the analysis (Flucke *et al.*, 2020 ▸) or connecting an external application via a bridge (Fangohr *et al.*, 2018 ▸). The DSSC detector produces up to 800 images per train with a data rate of 1 GB s^−1^ for a single module. To ensure low-latency data processing, we have used *EXtra-metro* (Schmidt, 2022 ▸), a framework developed in-house with intrinsic parallelization, which enables fast and reliable online previews of various analysis routines. These routines are generated by interpreting a Python script, where the analysis procedures are described.

During the experiment, the detector images are recorded while scanning either the X-ray photon energy or the pump laser time delay parameters. These data are simultaneously collected by *EXtra-metro* directly from the control system using the data pipelines for large detector data and from the central messaging broker for the control data. The analysis routines defined in the *SCS toolbox* package (Section 3.6) are then applied to the received data, using the pre-calculated non-linear and flat-field corrections, in a train-by-train manner.

**Figure 8:**
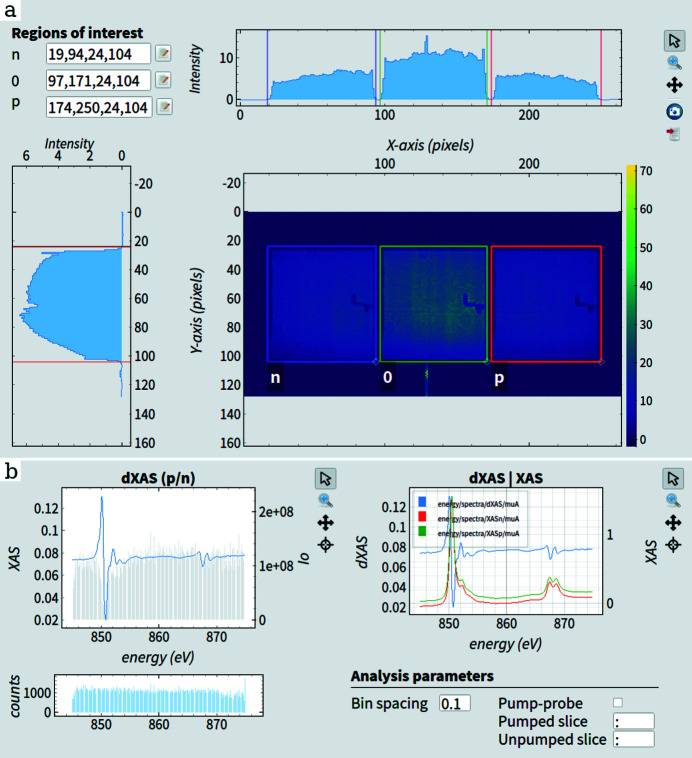
(*a*) Dark-corrected single-train image of the DSSC sensor in the Karabo GUI. The overlayed regions of interest define the intensity of the imaged beams and can be modified using the *EXtra-metro* parameter fields or by user interaction in the GUI. The projections along *x* and *y* axes are also plotted as guides for optimal zone plate alignment. (*b*) XAS spectra of the three ratios after an energy scan displayed in the Karabo GUI. The panel also contains the analysis parameter fields that can be changed during run time.

The processed data are then displayed in the Karabo graphical user interface. Custom widgets are developed for further data visualization and interaction using the GUI extensions (Flucke *et al.*, 2020 ▸). Fig. 8 ▸(*a*) shows a dark-corrected detector module with three overlayed ROIs that define the intensities of the pumped, unpumped and reference signals. These ROIs, along with other analysis parameters such as bin spacing and pulse selection, can also be modified during the run time. In addition to showing the DSSC image and ROIs, the vertical and horizontal projections of the intensity are displayed and are used during the initial zone plate alignment to find the center of the X-ray beam. Finally, the resulting X-ray absorption spectra of the signals are shown in Fig. 8 ▸(*b*) and are further discussed in the next section.

## 4. Results

### 4.1. Transient XAS

It is essential to verify the effect of the different levels of data correction on actual time-resolved data. To demonstrate this, we selected an extended XAS spectrum measured on a NiO thin film sample at the Ni *L*_3,2_ edge. The sample was excited above its band gap by an optical laser pulse with 266 nm wavelength, 50 fs pulse duration and 5 mJ cm^−2^ peak fluence. The time delay between optical pump and X-ray probe was fixed to 1.0 ps. During the FEL train, 18 pairs of optical pump/X-ray probes were used, with 17.8 µs separation between them, corresponding to an effective repetition rate of 56 kHz during the train.

**Figure 9:**
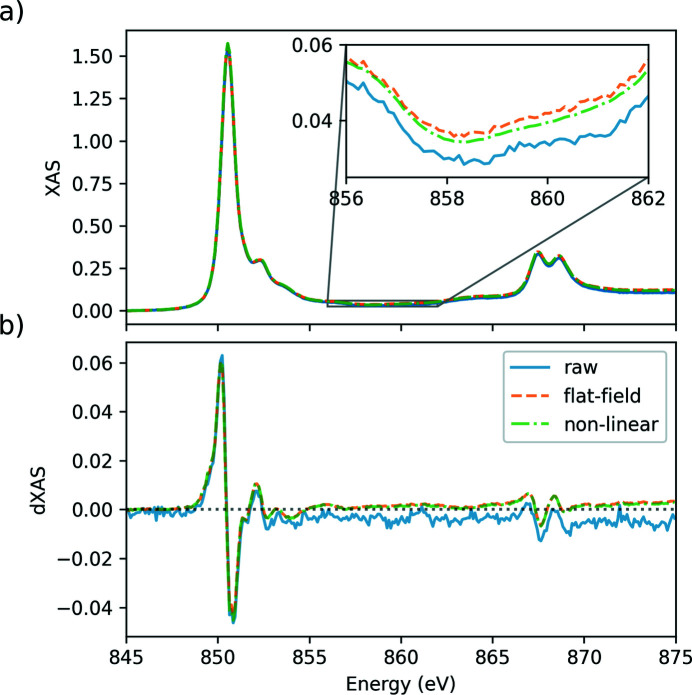
(*a*) XAS and (*b*) transient change in XAS at the Ni *L*_3,2_ edges of a NiO thin film. The data were recorded at a fixed time delay of 1.0 ps, with a peak fluence of 5 mJ cm^−2^ and a pump wavelength of 266 nm. Data that are only dark-corrected are shown as continuous blue lines and labeled ‘raw’. Data that are additionally flat-field corrected are shown as dashed orange lines and labeled ‘flat-field’. Data that are also corrected for non-linearity are shown as dot-dashed green lines and labeled ‘non-linear’.

In Fig. 9 ▸(*a*) the XAS and in Fig. 9 ▸(*b*) the pump-induced change in XAS at the Ni *L*_3,2_ edges are shown as continuous blue lines and labeled as ‘raw’ for data that are only dark-corrected. The energy range of these spectra spans 30 eV and is divided in 300 bins of 0.1 eV width. The data were collected for 30 min, which corresponds to 6 s per bin. With 10 trains per second at the European XFEL, and 18 X-ray pulses per train for this measurement, we collected a thousand X-ray pulses per bin. This provides a noise reduction of 30 which, together with a single-shot SNR of 300, gives a final noise of 10^−4^ which is necessary to record the smaller features in the transient XAS signal shown in Fig. 9 ▸(*b*). Discussions of the physics behind these NiO XAS and transient XAS are beyond the scope of this article and will be published separately. However, we can briefly discuss the XAS and transient XAS measured. The XAS is characteristic of NiO and its multiplet structure (Regan *et al.*, 2001 ▸; de Groot *et al.*, 2021 ▸) and comparable with the spectrum measured at Synchrotron SOLEIL (Rothenbach, 2020 ▸). In the simplest picture, the transient XAS probes the holes left by excited electrons after inter­action with the pump laser and is observed as an increase in absorption. At the same time, the states filled by excited electrons give a reduction of the XAS (Stamm *et al.*, 2007 ▸; Boeglin *et al.*, 2010 ▸; Willems *et al.*, 2020 ▸; Hennes *et al.*, 2020 ▸; Le Guyader *et al.*, 2022 ▸).

The amplitude of the transient XAS is of the order of 5% of the static XAS at most, as seen in Fig. 9 ▸(*b*). Although small, it is above the noise in the measurement even for the ‘raw’ data that are only dark-corrected. Here, we define noise as the apparent random fluctuations in data points that are close to each other. The situation improves drastically using the flat-field corrected data, which are shown as a dashed orange line in Fig. 9 ▸. Over the whole spectrum, the noise level in the transient XAS is significantly reduced compared with the uncorrected data. In the XAS spectra, this reduction is also visible in the inset in Fig. 9 ▸(*a*), which shows a zoomed region on the flat continuum transitions part of the XAS between the *L*_3_ and *L*_2_ edges. We note that, for this dataset, the characterization run without sample in the beam was not recorded at the time. To compute the flat-field and non-linear corrections, we instead selected in the data the shots falling in the flat pre-edge region below 848 eV. We do not expect the analysis to be significantly affected by this, as confirmed in experiments conducted later. The data, which are in addition corrected for non-linearity, are shown as a dot-dashed green line in Fig. 9 ▸. For the XAS, a further improvement in the data is visible in the zoomed inset, where the curve is now very smooth with negligible noise remaining. For the transient change, there is almost no visible difference between flat-field correction and non-linear correction. This is what one would expect from the results shown in Fig. 5 ▸, where the improvement with the non-linear correction is limited for the −1st/+1st order, which directly probes the transient XAS change, while the improvement is much larger for the other two ratios probing the unpumped and pumped XAS spectra.

While it is evident that each additional correction improves the data quality with a significant noise reduction, there are also some systematic deviations. This is shown by the fact that the three different levels of corrections do not result in curves overlapping each other in Fig. 9 ▸. Therefore, we have to discuss the implications of each correction on the data. The flat-field correction ensures that the measured intensity is independent of the FEL intensity profile impinging on the zone plate. Clearly, the transmission of the sample should not depend on the pulse-to-pulse fluctuating FEL intensity profile; therefore, data that are not corrected by the flat field cannot be trusted. Similarly, the non-linear correction ensures that the measured quantity does not depend on the X-ray intensity, so data that are not corrected for the remaining DSSC non-linearity cannot be trusted either. Are there systematic variations in flat-field and non-linearity corrected data that require additional correction? There is one, which is visible in Fig. 9 ▸(*b*), where the baseline of the transient XAS seems to shift away from zero. This is probably related to the flat-field correction, which is calculated at a fixed photon energy. However, the zone-plate properties depend on the photon energy. This can be corrected with an additional step, where we record an XAS spectrum without sample, from which the linear background can be extracted and subtracted from flat-field and non-linearity corrected data. Such data are not available for this particular dataset, so this correction cannot be applied here. It is, however, now part of the standard measuring protocol.

Overall, we have shown that the different data corrections allow the extraction of the most information from every photon detected and the recording of XAS and transient XAS with excellent SNR, approaching the photon shot-noise limit.

### 4.2. Setup limits

As we have shown, the BOZ setup at SCS allows recording XAS with sensitivity reaching the photon shot-noise limit. Detecting more photons or increasing the repetition rate of the experiment are two simple means by which we can increase the statistics. In this section, we thus discuss the limits in terms of X-ray photons that we can use and the limits in terms of repetition rate. Finally, given these limits, we review which sample systems can be measured with this setup.

#### 4.2.1. X-ray fluence

If our setup is photon shot-noise limited, then counting more photons by increasing the beam intensity would directly translate into a better signal with less noise, as confirmed by Fig. 7 ▸. This is true until we reach saturation of pixels in the DSSC detector too frequently, as such data have to be discarded from the analysis, thereby reducing the final statistics. In practice, the X-ray intensity is adjusted such that few percents of the shots are saturated. If the intensity of the monochromatic X-ray could be made more stable, for example by employing a self-seeding scheme (Serkez *et al.*, 2013 ▸), frequent pixel saturation could be avoided while collecting intense shots more regularly, resulting in higher final photon counts. Given this pixel saturation and the lack of available soft X-ray seeding scheme at SCS, the only way to increase the intensity would be to enlarge the beam even more on the DSSC sensor. Here, we reach two limits with the current setup. First, expanding the beam even more means that we need to either move the DSSC further downstream or use a BOZ with a shorter focal length. However, the DSSC detector is already placed as far downstream as possible given the current size of the SCS experiment hutch. Using a BOZ with a shorter focal length would reduce the space available between the BOZ and the sample to couple in the optical pump laser. Moreover, given that, with the current setup, we are nearly fully illuminating the sensor, as seen in Fig. 4 ▸, expanding the beam further would require a larger monolithic sensor. Clearly, given that the DSSC detector is currently the only detector capable of recording up to 800 pulses per train at 4.5 MHz repetition rate that can be delivered by the European XFEL at SCS (Porro *et al.*, 2021 ▸), we are at the limit. We could only make use of higher beam intensity with a new detector having higher saturation limits, a larger continuous sensor, or smaller pixels with similar electron well depth.

**Figure 10:**
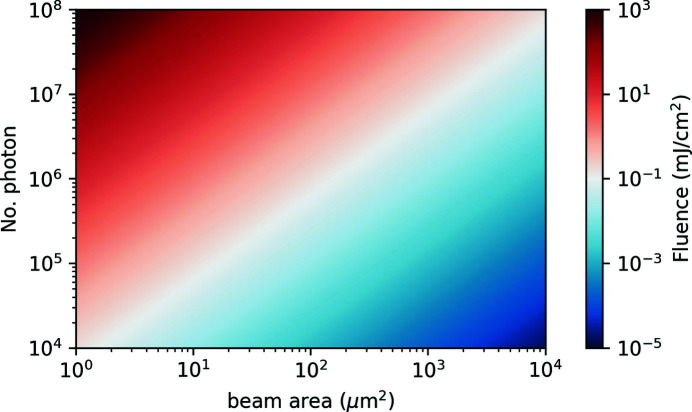
X-ray fluence on the sample, in mJ cm^−2^, as a function of the beam size and the number of 1 keV photons in the beam. A limit of 0.1 mJ cm^−2^, ensuring negligible non-linear X-ray absorption effects, is shown as white color. Values below and above this limit are shown in blue and red, respectively.

However, detecting more photons is not the only aspect we should discuss here. With increasing the X-ray intensity, eventually non-linear X-ray absorption effects will set in, where the X-ray pulse modifies the sample that it probes (Wu *et al.*, 2016 ▸; Higley *et al.*, 2019 ▸). In Fig. 10 ▸, we show the X-ray fluence as a function of the X-ray spot size on the sample and the number of photons in the beam for photons with 1 keV energy. Non-linear X-ray phenomena set in at fluences of a few mJ cm^−2^ (Wu *et al.*, 2016 ▸; Higley *et al.*, 2019 ▸), so we need to stay below 0.1 mJ cm^−2^ to be on the safe side. With a typical beam size of 30 µm × 30 µm, the beam intensity should stay below a few 10^6^ photons. This is already the range we reached, as shown in Fig. 5 ▸, so we are close to the limit here as well. It is possible, in principle, to increase the X-ray beam size on the sample by simply moving it further downstream of the zone-plate focus. However, a larger X-ray beam size means an even larger optical pump spot size, which results in slower heat dissipation. As discussed in the next section, slow heat dissipation limits, in turn, the number of X-ray pulses that can be used per train such that the beneficial effect of increased statistics per shot with increased spot size might be compensated by a reduced number of shots due to sample heating.

#### 4.2.2. Repetition rate

In stroboscopic pump–probe experiments, heat dissipation is a known issue limiting the effective repetition rate at which data can be collected. This is particularly the case here, as the samples are X-ray transparent thin membranes, which limit the heat dissipation to the two in-plane dimensions. Moreover, the X-ray pulse pattern at the European XFEL, with its trains of X-ray pulses at up to 4.5 MHz, leaves very limited time between the X-ray pulses in the train for heat dissipation to take place.

**Figure 11:**
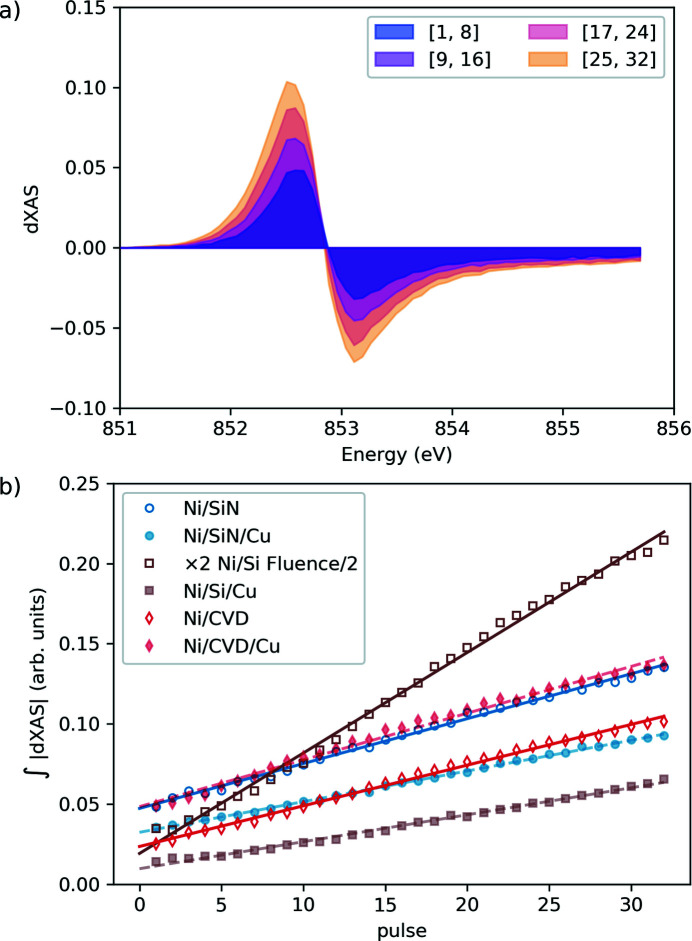
(*a*) Transient change in XAS in the Ni/Si_3_N_4_ sample as a function of photon energy for selected pulses in the train as given by the pulse range in the corresponding legend. The time delay was 0.5 ps, the laser fluence was 7 mJ cm^−2^, and the pump wavelength was 800 nm. The repetition rate of the 32 pulses during the FEL train was 280 kHz. (*b*) Integral of the absolute change in XAS as a function of the pulse number in the train, ranging from 1 to 32. The points are the measured data, while the lines are linear fits.

In Fig. 11 ▸(*a*), the transient change in XAS is shown as a function of the photon energy around the Ni *L*_3_ absorption edge in a 20 nm Ni sample for different ranges of optical pump/X-ray probe pulse pairs within each train. A clear trend is visible, where the change in XAS increases with the pulse pair number. In a simple picture, we can interpret this change in XAS as a change in electron population around the Fermi level. The integral of the absolute change in XAS over the spectral range measured is then proportional to the deposited energy in the system. We plot this deposited energy as a function of the pulse pair number in the train, for different sample stacks, as shown in Fig. 11 ▸(*b*). The same Ni film is deposited on membranes made of silicon, silicon nitride and diamond, with and without a Cu heat sink for each case. The diamond membranes are prepared by chemical vapor deposition (CVD). All data in Fig. 11 ▸ appear to be linear, *i.e.* each optical pump pulse in the train adds energy to the system that does not dissipate completely before the next pulse arrives, leading to a temperature increase of the sample. We fitted these data with a straight line and extracted the slope and intercept, as listed in Table 1 ▸. In the ideal case, the sample would be efficiently excited with the first pump pulse, meaning that we would measure a large intercept. At the same time, the sample would cool down efficiently until the next pump pulse arrives, meaning that we would measure the same excited sample for subsequent pulses without any heat accumulation, resulting in the slope being zero. Therefore, an optimal sample stack combines a large intercept and a small slope. To characterize this property of the sample stack, we introduce a figure of merit as the ratio of the intercept and the slope. The larger this number, the better suited the sample stack is for repetitive pump–probe experiments within the train. Looking at Table 1 ▸, we can see that the worst sample would be the Si substrate. Indeed, for this sample stack, we had to reduce the fluence by a factor of two, otherwise the sample would break, suggesting that heat accumulation in this sample is strong. For the Si_3_N_4_ sample, the presence of the Cu heat sink does not improve the performance, but both are some of the best sample stacks. For the CVD diamond substrate, the Cu heat sink improves the performance by a factor of two but does not perform better than Si_3_N_4_-based stacks.

In all the cases presented here, the heat accumulation is a measurable effect on top of the transient change in XAS. If no further analysis is possible, we are forced to limit the number of pulses used in the experiment and separate them as far as possible, given the European XFEL pulse pattern, which in practice is often 10 to 20 pulses per train. However, if one can assume the response of the system to be linear both on the transient change and on the heat accumulation change, then the two contributions can be disentangled and possibly more pulses per train can be used with improved statistics.

Having discussed the limits in terms of X-ray fluence and repetition rate in the previous section, we now discuss from which classes of sample systems can we expect a measurable signal level.

#### 4.2.3. Sensitivity limits

To make the best use of the setup and the detected photons, the intensity of the three beams on the DSSC should be similar. This way, the full dynamic range of the DSSC pixels can be used, from the dark pedestal level up to the saturation level or to the 4.5 MHz digitization cutoff. If the three beam intensities are not well balanced, the efficiency of the setup will be reduced, as already discussed with equation (9). In the case of a strongly absorbing sample at a resonant edge, the measurement will be limited both by saturation of the DSSC detector on the pre-edge region and by low transmitted intensity at the resonance. In practice, these considerations limit the sample thickness to one or two absorption lengths at most.

In other cases, one might be interested in systems that are much more diluted. These are the cases considered in Table 2 ▸. We begin with the case of a 20 nm-thick Fe film, which corresponds to one absorption length at the *L*_3_ resonance. The static XAS signal is thus 1, as shown in Table 2 ▸. For the noise level, we can estimate it to be similar to a no-sample case, as the change in the number of detected photons will be moderate. This gives us a single-shot SNR of 250. For the other cases that we consider, the absorption is smaller, so the number of detected photons, and therefore the noise, will be constant. The only thing that changes is the level of signal. For example, for this 20 nm Fe film, we can roughly estimate the transient XAS to be around 5% of the static XAS. In other words, the signal reduces by a factor of 20, giving a single-shot SNR of 12, as shown in Table 2 ▸. Ideally, we would like to measure the transient XAS with an overall SNR greater than 3, which can be achieved in this case with a single shot, as indicated in the last column of Table 2 ▸.

If we now consider the case of an Fe monolayer, the static XAS signal scales down by a factor of 50. This results in a single-shot SNR of 5 for the XAS and 0.2 for the transient XAS. To achieve an SNR of 3 on the transient XAS, we have to average 225 pulses. This is between 22.5 s of data acquisition if the sample can only be pumped at 10 Hz and 1 s and if the sample can be pumped with 20 shots during one FEL train.

If we now consider the case of a single layer of molecules containing a single Fe central atom, we would have tens to hundred times less absorbing atoms than in the Fe monolayer case, which means hundreds to tens of thousands more shots and longer acquisition required to reach the target SNR, making this kind of experiment challenging even with many pulses per train.

Finally, we have described in this article the case of a homogeneous sample, where both the excited and unexcited sample membranes can be prepared identical. In practice, samples are often inhomogeneous, such that the transient XAS computed from the excited and unexcited membrane might not be meaningful. In such cases, the transient XAS can be measured with this setup using the excited and reference membranes, by alternating pumped and unpumped shots during the FEL train. The benefits of photon-shot-noise-limited detection and shot-by-shot normalization remain.

## 5. Conclusion

The beam-splitting off-axis zone plate setup, which is available to users at the SCS instrument at the European XFEL, was presented in detail. We showed that two essential data correction steps are necessary to make the best use of the collected photons: a flat-field normalization that compensates for the inhomogeneous diffraction efficiency of the diffractive optics employed, and a correction of the remaining DSSC non-linearity. Remarkably, with these two corrections, the resulting data are shown to be close to the photon shot-noise limit. In addition, we reviewed several tools that we provide to the users, namely a beam propagation calculator to help users design their samples to be compatible with the fixed beam position of this setup, the complete analysis procedure in the form of a Python package and associated *Jupyter* notebooks, and finally the online analysis framework to process live the measured data with all the levels of correction available. We showed an example of transient XAS in NiO at the Ni *L*_3,2_ edges with unprecedented data quality, and discussed the effect of the different corrections. Finally, we reviewed the current limits of the existing setup in terms of the number of photons on the sample, the repetition rate that thin X-ray transparent samples can accommodate, and the signal level for increasingly dilute systems. We finally conclude that the current setup is as good as it can be with the current detector, and that we can measure transient XAS down to a few tens of layers of molecules.

Further improvement in the sensitivity of the setup may come from the implementation of a self-seeding scheme, or by using an improved detector, such as the DEPFET DSSC, featuring a lower readout noise and higher dynamic range than the current MiniSDD DSSC we used here. With the installation of the Apple-X helical afterburners in the near future, circular- and linear-polarization-dependent transient XAS experiments, such as X-ray magnetic circular dichroism and X-ray magnetic linear dichroism, will become possible. Plans to adapt this setup to flat liquid jet experiments to investigate, for example, optically driven transitions in molecules in solution are ongoing. Finally, we note that the data processing that we presented provides a clear path for a data reduction strategy that can be applied on the fly, as the needed data corrections are computationally fast. This means that this method is compatible with quasi-continuous operation at several tens to hundreds of kHz. Such a regime will become available with forthcoming FEL accelerators that are being developed and would be ideal for experiments on X-ray transparent thin solid samples.

## 6. Data availability

All data are available in the main text or the supporting information. The raw data generated at the European XFEL for the experiment UP2161 used in Fig. 11 ▸ are available at https://doi.org/10.22003/XFEL.EU-DATA-002161-00. For the experiment UP2712 used in Figs. 4 ▸, 5 ▸, 6 ▸ and 7 ▸, raw data are available at https://doi.org/10.22003/XFEL.EU-DATA-002712-00. For the experiment UP2589 used in Figs. 1 ▸ and 9 ▸, raw data are available at https://doi.org/10.22003/XFEL.EU-DATA-002589-00.

## Supplementary Material

European XFEL for the experiment UP2161: https://doi.org/10.22003/XFEL.EU-DATA-002161-00

European XFEL for the experiment UP2712: https://doi.org/10.22003/XFEL.EU-DATA-002712-00

European XFEL for the experiment UP2589: https://doi.org/10.22003/XFEL.EU-DATA-002589-00

## Figures and Tables

**Table 1:** Slope and intercept and their ratio (intercept/slope) fitted from the integral of the absolute change in XAS as a function of the pulse number in the train shown in Fig. 11 ▸ The samples are 20 nm Ni film capped with 2 nm MgO and grown on different sample stacks listed below with thickness in nm.

Membrane	Heat sink	Slope (10^−3^)	Intercept (10^−2^)	Ratio
Si_3_N_4_(200)	—	2.8	4.7	16.9
Si_3_N_4_(200)	Cu(100)	1.9	3.2	16.9
Si(200)	—	6.3	1.9	3.0
Si(200)	Cu(100)	1.7	1.0	5.7
CVD diamond(100)	—	2.6	2.3	9.3
CVD diamond(100)	Cu(100)	2.9	4.8	16.6

**Table 2:** Single-shot static and transient XAS signal as well as SNR for different cases To achieve an SNR greater than 3 for the transient XAS signal, the minimal number of shots is given in the last column.

	Static		Transient		
Sample	Signal	SNR	Signal	SNR	Minimal shots
Fe (20 nm)	1	250	0.05	12	1
Fe monolayer (0.287 nm)	2 × 10^−2^	5	10^−3^	0.2	225
Molecule on surface	10^−3^–10^−4^	0.25–0.025	5 × 10^−5^–5 × 10^−6^	10^−2^–10^−3^	10^5^–10^6^

## Footnotes

1 DSSC stands for DEPFET sensor with signal compression, with DEPFET standing for depleted p-channel field-effect transistor. However, the DSSC detector that was employed in this article is using a mini-silicon drift detector (MiniSDD) sensor and is described in detail by Porro *et al.* (2021 ▸). The two cameras (MiniSDD based and DEPFET based) share the same readout electronics and geometry.
